# Integrated Bioinformatics-Based Identification of Ferroptosis-Related Genes in Carotid Atherosclerosis

**DOI:** 10.1155/2022/3379883

**Published:** 2022-11-07

**Authors:** Linfeng Wei, Nan Wang, Runsheng Li, Hui Zhao, Xin Zheng, Zhihui Deng, Zhongwei Sun, Zhuangjie Xing

**Affiliations:** ^1^Department of General Surgery, Zhongshan Hospital of Dalian University, Dalian, 116001 Liaoning, China; ^2^Eye Center of Xiangya Hospital, Central South University, Changsha, 410008 Hunan, China

## Abstract

**Background:**

Ferroptosis, a type of cell death caused by phospholipid peroxidation, has lately been linked to the onset and development of numerous illnesses. Numerous investigations have demonstrated the close relationship between lipid peroxidation and carotid atherosclerosis. In order to get new knowledge for targeted therapy, bioinformatics analysis was employed in this study to discover the probable ferroptosis-related genes of carotid atherosclerosis.

**Methods:**

The GSE43292 gene expression dataset was downloaded from the Gene Expression Omnibus (GEO) database. The differentially expressed ferroptosis-related genes were screened by R software and then analyzed by protein-protein interaction (PPI) network, differential gene correlation analysis, Kyoto Encyclopedia of Gene and Genome (KEGG) pathway, and Gene Ontology (GO) terminology enrichment analysis to explore the functional role.

**Result:**

In samples of atherosclerosis, we found 33 ferroptosis genes that were differentially expressed, including 21 upregulated genes and 12 downregulated genes. These differentially elevated genes were mainly connected to the ferroptosis and glutathione metabolism pathways, according to GO and KEGG enrichment analysis. We also discovered 10 hub genes and 2 important modules through the analysis of the PPI network and the creation of key modules.

**Conclusion:**

The current findings imply that the carotid atherosclerosis phenomenon involves ferroptosis, and 10 important genes associated to ferroptosis may play a role in the development of carotid atherosclerosis. This study offered a novel approach to future research on the carotid atherosclerosis pathogenic processes and treatment targets.

## 1. Introduction

Carotid atherosclerosis is closely associated with an increased risk of atherosclerotic cardiovascular disease and ischemic strokes, the third largest cause of mortality and the largest cause of disability [1, 2]. Studies have revealed that plaque formation in coronary arteries and carotid arteries has a similar pathological basis, and noninvasive examination of carotid artery-related indicators can effectively predict the risk of coronary heart disease and intervene in a timely manner, which is important for reducing the incidence of cardiovascular events in patients with coronary heart disease [3]. Meanwhile, 18-25% of thrombo-embolic strokes are caused by carotid atherosclerosis [4]. Despite the fact that carotid atherosclerosis can be treated with successful carotid endarterectomy to prevent arterial stenosis and lessen the risk of ischemic stroke, about 6% of patients who have the procedure are still at risk for stroke, making it the third leading cause of death and the main cause of disability [5]. Therefore, further research into the causes of carotid atherosclerosis is needed to provide novel perspectives on how to effectively treat it and stop it from progressing. Previous research has demonstrated that a number of variables, such as advanced age, gender, a history of long-term smoking, hypertension, diabetes, and hyperlipidemia, might impact carotid atherosclerosis [6–12]. Oxidative stress is well acknowledged to have a significant part in the etiology of carotid atherosclerosis, which involves complex pathways. Reactive oxygen species (ROS), including superoxide, hydroxyl, and perhydroxyl radicals, can activate a lot of proinflammatory genes, cause functional damage to many components, and ultimately end in cell death [13, 14].

Ferroptosis, a novel form of programmed cell death distinct from apoptosis and necrosis, is characterized by lethal accumulation of lipid peroxides [15–18]. In the presence of divalent iron or ester oxygenase, the major mechanism of ferroptosis is to catalyze the high production of unsaturated fatty acids on the cell membrane, which results in lipid peroxidation and causes cell death. And ferroptosis is characterized by a decline in the glutathione system's regulatory core enzyme glutathione peroxidase 4 (GPX4), which is primarily mediated by the system xc-/glutathione (GSH)/GPX4 axis. As a powerful reductant and cofactor of GPX4, GSH is depleted as a result of system xc- inhibition. Therefore, GSH inhibits the activity of GPX4, which in turn encourages the generation of ROS and results in ferroptosis [16, 19]. Recently, studies have shown that ferroptosis is involved in the occurrence and development of carotid atherosclerosis [20]. As an effective inducer of ferroptosis, erastin downregulates GPX4 and upregulates the expression of acyl-coA synthetase long chain family member 4 (ACSL4) to promote ferroptosis in endothelial cells during atherosclerosis, while miR-17-92 protects endothelial cells from ferroptosis induced by erastin by targeting the A20-ACSL4 axis [21]. Studies on atherosclerosis have discovered that nontransferrin bound serum iron (NTBI) excess might hasten the progression of atherosclerosis by triggering vascular cell death, vascular endothelial cell activation, and a significant increase in MCP-1-mediated monocyte recruitment [22]. A HFD-fed ApoE−/- mice study showed that inhibition of ferroptosis could protect against lipid peroxidation and the aggravation of atherosclerosis [23]. In addition, Zhou et al. found the expression of Ptgs2 and ACSL4 was upregulated, while GPX4 was downregulated in the advanced stages of atherosclerosis [24]. To provide novel perspectives on the prevention and treatment, more potential target and ferroptosis-related genes in carotid atherosclerosis should be further explored.

Our study used the Ferrdb Database (http://www.zhounan.org/ferrdb/le(3)gacy/index.html) and analyzed the microarray dataset GSE43292, which contains carotid atherosclerotic plaques and normal tissue samples, created by Bricca et al. First, we analyzed differentially expressed ferroptosis-related genes in carotid atherosclerosis by the “limma” package. Subsequently, we conducted correlation analysis, GO and KEGG analysis, and protein-protein interaction (PPI) network analysis for these differentially expressed ferroptosis-related genes and ultimately identified 10 key genes. In summary, our findings provide novel perspectives on the clinical diagnosis and management of carotid atherosclerosis and contribute to our understanding of the function of ferroptosis in carotid atherosclerosis.

## 2. Materials and Methods

### 2.1. Data Acquisition

The microarray expression profiling dataset GSE43292 was downloaded from Gene Expression Omnibus (GEO, https://www.ncbi.nlm.nih.gov/geo/), a platform to collect high-throughput sequencing and microarray-based sequencing data. GSE43292 is in GPL6244 platform (Affymetrix Human Gene 1.0 ST Array), which included 32 carotid atherosclerotic plaque tissue samples and 32 control tissue samples. A total of 506 ferroptosis-related genes were downloaded from the Ferrdb Database (http://www.zhounan.org/ferrdb/legacy/index.html).

### 2.2. Differential Expression Analysis

The normalized expression matrix was obtained from the microarray expression profile dataset GSE43292 by using R software (version 4.0.1). Then, the probe in the dataset was annotated by annotation file. We obtained the differentially expressed ferroptosis-related genes by using the “limma” package in R software, and the threshold was set to adjusted *P* < 0.05 and log FC > 0.5.

### 2.3. Correlation Analysis of Differentially Expressed Genes (DEGs)

The “corrlot” package of R software was used to evaluate the ferroptosis-related differentially expressed genes and reveal the connection between differentially expressed genes.

### 2.4. GO and KEGG Pathway Enrichment Analysis of Differentially Expressed Ferroptosis-Related Genes

Enrichment analysis of Gene Ontology (GO) terms and Kyoto Encyclopedia of Genes and Genomes (KEGG) pathways were analyzed by using “clusterProfiler” package in R software. The GO analysis includes three categories: cell composition analysis (CC), biological process analysis (BP), and molecular function analysis (MF).

### 2.5. Protein-Protein Interaction (PPI) Network

PPI network analysis was analyzed by STRING database (https://string-db.org/), which can query and predict protein-protein interactions. The visualization of the PPI network was constructed by the Cytoscape software (version 3.6.0).

## 3. Results

### 3.1. Identification of Differentially Expressed Ferroptosis-Related Genes

**Figure 1:**
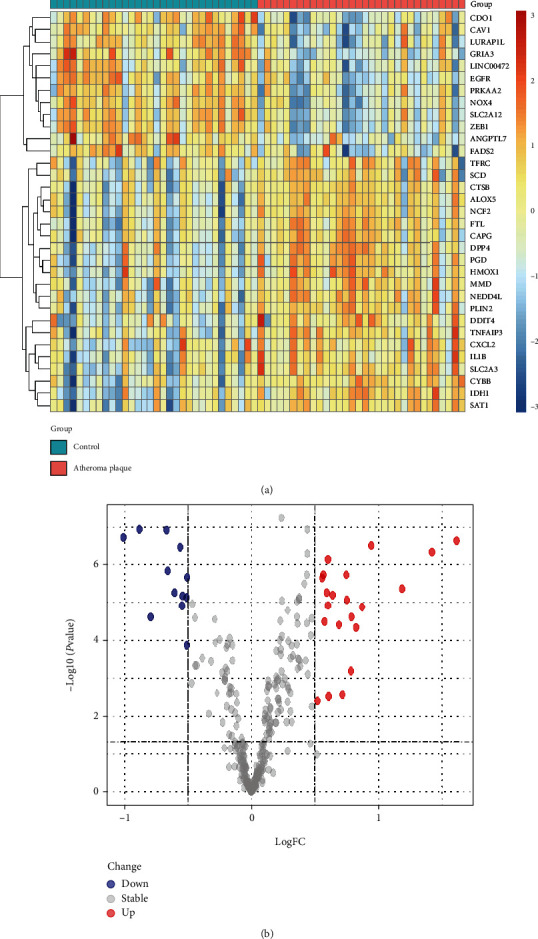
Differentially expressed ferroptosis-related genes in carotid atherosclerotic plaque tissue samples and control samples (normal). (a) Heat map of the 33 differentially expressed ferroptosis-related genes in atheroma plaque samples and healthy samples. (b) Volcano plot corresponding to the GSE43292 dataset. The red dots represent the significantly upregulated genes, and the blue dots indicate the significantly downregulated genes.

**Figure 2:**
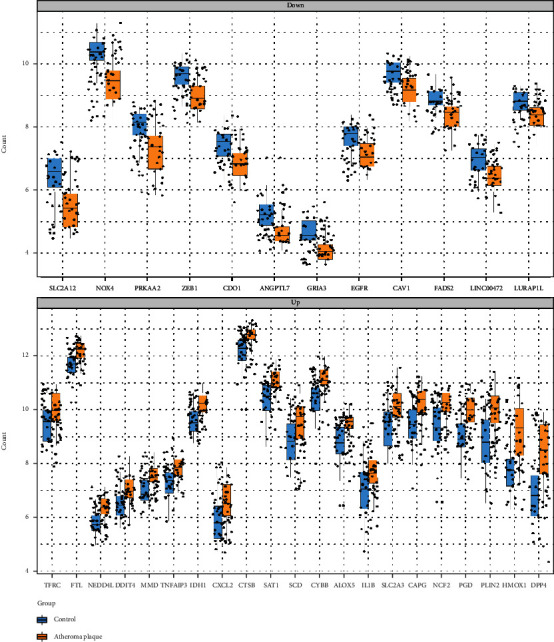
The boxplot of 33 differentially expressed ferroptosis-related genes in plaque tissue samples and normal samples.

The microarray expression profile dataset GSE43292, which included 32 carotid atherosclerotic plaque tissue samples and 32 control tissue samples, was downloaded from GEO. Then, we analyzed the expression of 506 ferroptosis-related genes obtained from the Ferrdb Database in the GSE43292 dataset. Then using the threshold of adjusted *P* value <0.05 and log FC > 0.5, we obtained a total of 21 upregulated and 12 downregulated genes (Table 1). The 33 differentially expressed genes between carotid atherosclerosis plaque and control group obtained above were processed by R software and displayed in the form of heat map and volcano plot (Figures 1(a) and 1(b)). Moreover, box plots showed 33 differentially expressed ferroptosis-related genes (Figure 2); the top five upregulated genes were DPP4, PGD, HMOX1, IDH1, and CYBB; and the top five downregulated genes were NOX4, ZEB1, SLC2A1, GRIA3, and CDO1.

In Table 1, these 33 genes are differentially expressed ferroptosis-related genes in carotid atherosclerosis analyzed by R software, and among them, HMOX1, NCF2, ALOX5, CYBB, IL1B, TFRC, NOX4, EGFR, and CAV1 have strong relevance in the carotid atherosclerosis and ferroptosis.

### 3.2. Correlation of Differentially Expressed Ferroptosis-Related Genes

**Figure 3:**
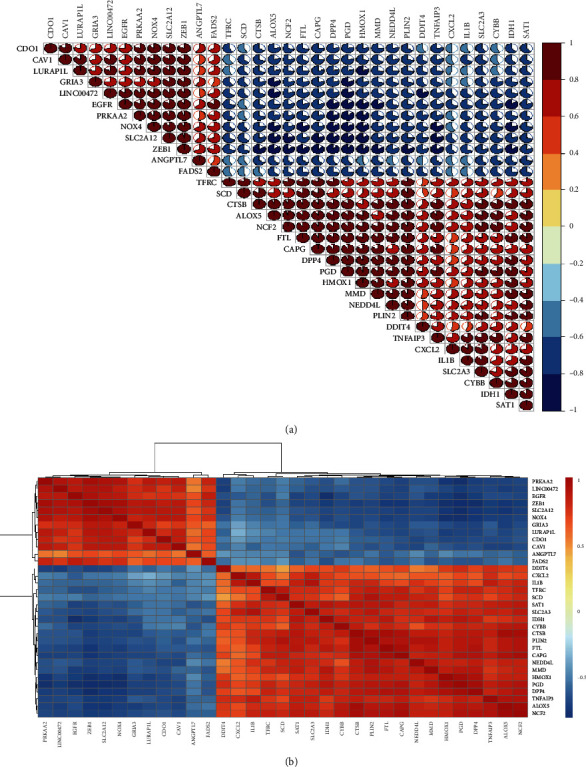
Spearman correlation analysis of 33 differentially expressed ferroptosis-related genes.

To explore the expression correlation of these ferroptosis-related genes, we employed the correlation analysis, the results showed the correlation of the whole 33 differentially expressed genes, and there existed a high correlation between upregulated genes and the same between downregulated genes (Figures 3(a) and 3(b)).

### 3.3. Functional and Pathway Enrichment of the Differentially Expressed Ferroptosis-Related Genes

**Figure 4:**
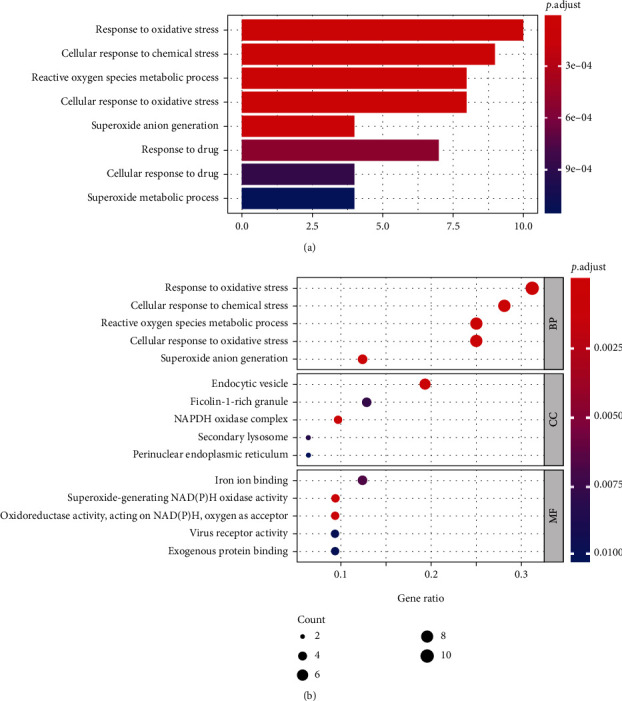
Gene Ontology (GO) enrichment analysis of 33 differentially expressed ferroptosis-related genes. (a) Bar plot of enriched GO terms. (b) Bubble plot of enriched GO terms. Abbreviations: BP: biological process; CC: cellular component; MF: molecular function.

**Figure 5:**
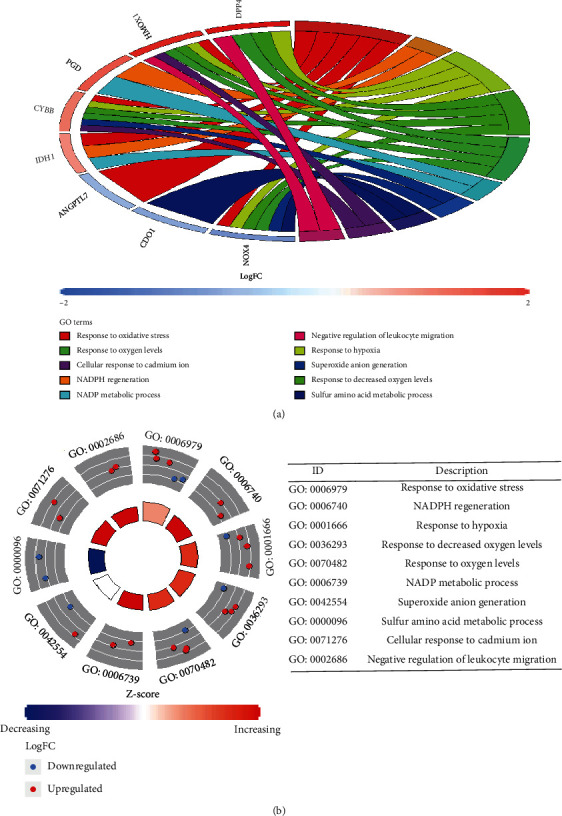
Gene Ontology (GO) enrichment analysis of 33 differentially expressed ferroptosis-related genes. (a) Chordal graph of enriched GO terms. (b) Eight diagrams of enriched GO terms.

**Figure 6:**
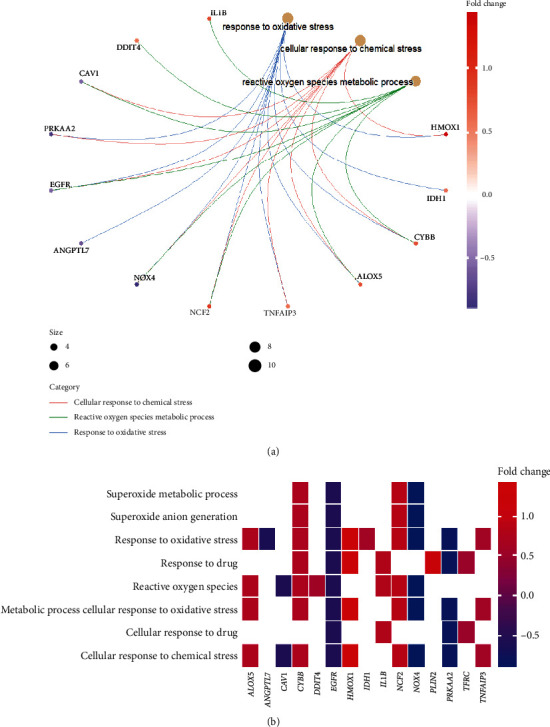
(a) Relationships between enriched pathways. (b) Heat map-like functional classification.

**Figure 7:**
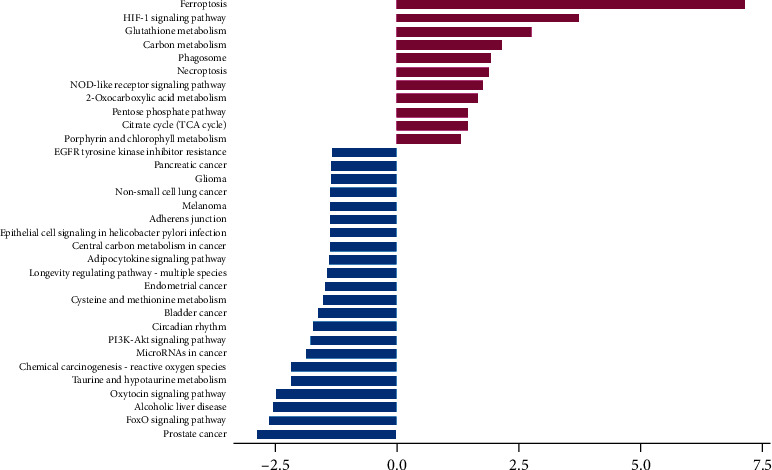
Kyoto Encyclopedia of Genes and Genomes (KEGG) enrichment analysis of 33 differentially expressed ferroptosis-related genes.

To investigate potential biological functions of these differentially expressed ferroptosis-related genes, we conducted Gene Ontology (GO) enrichment analysis and KEGG pathway analysis by using “clusterProfiler” package in R software (Table 2). The results revealed that the most significant GO enriched terms involved in oxidative stress, reactive oxygen species metabolic process, NADPH oxidase complex, iron ion binding, and others (Figures 4 and 5). Then, we analyzed 13 common genes of the three most significant pathways, namely, IL1B, DDIT4, CAV1, PRKAA2, EGFR, ANGPTL7, NOX4, NCF2, TNFAIP3, ALOX5, CYBB, HMOX1, and IDH1 (Figure 6(a)). Besides, the heat map-like functional classification map was used to show the enrichment of these genes in the most significant 8 pathways (Figure 6(b)). In KEGG enrichment analysis, the differentially expressed ferroptosis-related genes are mainly involved in the process of ferroptosis and glutathione metabolism (Table 2 and Figure 7).

### 3.4. PPI Network Analysis and Hub Gene Identification

**Figure 8:**
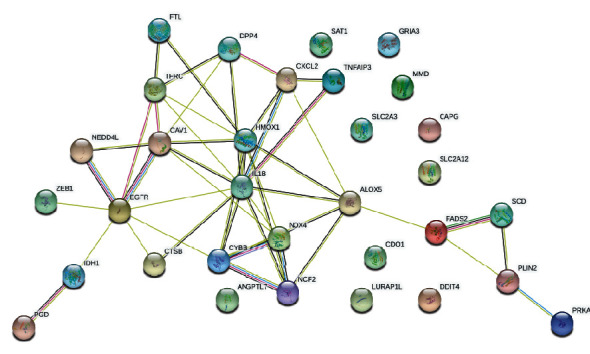
The DEG PPI network constructed using the STRING database.

**Figure 9:**
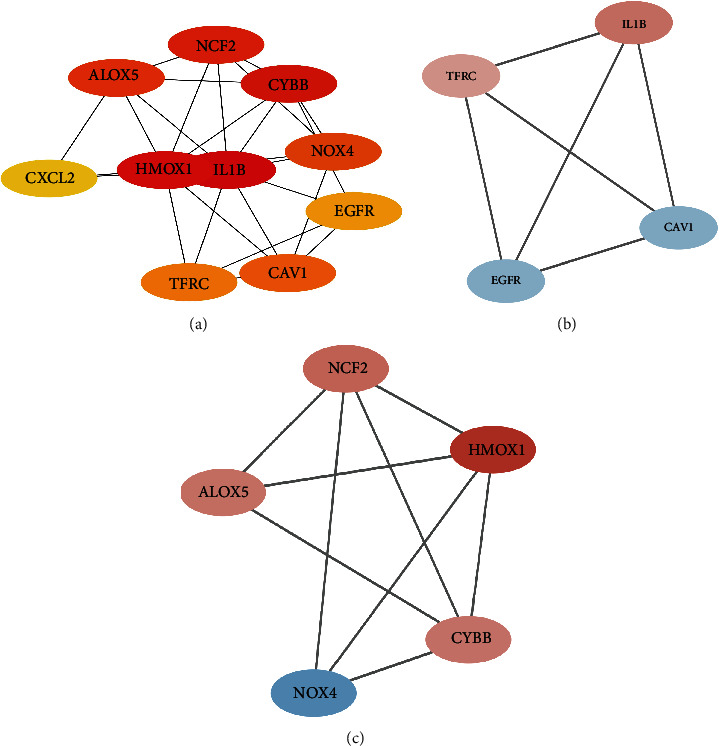
(a) The top 10 key genes were screened through the PPI network map. The nodes represent genes, and the edges represent links between genes. (b, c) Two key modules were identified by Cytoscape, which was used to identify network gene clustering.

The differentially expressed ferroptosis-related gene PPI network was constructed by using the Search Tool for the Retrieval of Interacting Genes (STRING) to determine the interactions among them (Figure 8). And the top 10 hub genes therein with the highest degree values were screened using Cytoscape (v. 3.9.0) (Figure 9(a)). Two key modules (Figures 9(b) and 9(c)) with six upregulated genes (HMOX1, NCF2, ALOX5, CYBB, IL-1B, and TFRC) and three downregulated genes (NOX4, EGFR, and CAV1) were identified.

## 4. Discussion

Carotid atherosclerosis is a chronic arterial disease characterized by the accumulation of lipid and fibrillar components in the arterial intima, associated with oxidative (ox-LDL) modification of LDL deposited into the intima, which is relevant to the process of carotid stenosis, insufficient cerebral blood supply, or local thrombosis [25]. These complications play an important role in the development of ischemic stroke [3, 26]. Ferroptosis is a way of regulated cell death mediated by intracellular iron accumulation, lipid peroxides, and the regulation of the system xc−/GSH/GPX4 axis [27]. The specific mechanism is that the cystine/glutamate antiporter system xc− increases intracellular glutamate and cystine by transmembrane transport. Intracellular cystine is reduced to cysteine and combines with glutamate to form GSH, which is a potent reductant and a cofactor for GPX4. As a member of the glutathione peroxidases, GPX4 reduces toxic lipid hydroperoxides (lipid-OOH) in cell membranes to nontoxic lipid alcohols (lipid-OH) by utilizing GSH as the electron donor to inhibit lipid peroxidation. SLC7A11, as a subunit of system xc−, can promote the generation of GSH. Therefore, the inhibition of SLC7A11 can inactivate GPX4 and lead to cell death [13, 17, 20, 28].

Recently, several studies have shown that ferroptosis, as an important pathway of lipid peroxidation, may promote the occurrence and development of carotid atherosclerosis. Ferroptosis can lead to rapid lipid peroxidation of cells due to lack of GPX4, resulting in vascular endothelial cell damage and accelerating the occurrence of carotid atherosclerosis [29]. In addition, ferroptosis induces lipid peroxidation of macrophages through NADPH oxidase4- (NOX4-) ROS-P38-MAPK signal pathway, which induces the production of foam cells and cause atherosclerotic plaque [30, 31]. The above studies can prove that ferroptosis plays an important role in carotid atherosclerosis. However, the mechanism through which ferroptosis initiates the development and progression of carotid atherosclerosis has not been clarified and need to be further explored.

In this study, we identified 33 differentially expressed ferroptosis-related genes in carotid atherosclerosis by bioinformatics methods. Some of these ferroptosis-related genes in the pathogenesis of carotid atherosclerosis has been previously studied [24, 32]. For example, TLR5-NOX4 signal cascades can stimulate proinflammatory cytokine expression through activation of NF-*κ*B or regulate smooth muscle cell migration through the TLR5-NOX4-RAC-JNK axis, which leads to the formation of neointimal plaques in atherosclerosis [33]. In addition, HMOX1 has been identified as a key ferroptosis-related gene in carotid atherosclerosis; however, it has not been thoroughly investigated yet [34, 35]. In our study, we will further investigate genes associated with ferroptosis in carotid atherosclerosis.

To explore the potential biological functions of these differentially expressed ferroptosis-related genes, we performed GO and KEGG enrichment analysis. The results showed that these genes were enriched for several terms such as ferroptosis, glutathione metabolism, and oxidative stress response, which were closely related to ferroptosis. Several studies have confirmed that ferroptosis can affect the progress of carotid atherosclerosis. A study has shown that ferrostatin-1 (Fer-1), a ferroptosis inhibitor, increases the expression of SLC7A11 and GPX4 by reducing the accumulation of iron in cells, thus decreasing the accumulation of lipid ROS, eliminating lipid peroxidation and endothelial cell damage, and inhibiting the development of carotid atherosclerosis [23]. We will further explore ferroptosis-related genes in carotid atherosclerosis in our study.

Based on the results of bioinformatics analysis, we further identified 10 most critical ferroptosis-related genes in carotid atherosclerosis by PPI analysis including HMOX1, IL1B, and NOX4. There is evidence suggested that some of these genes played an important role in the occurrence and development of ferroptosis and carotid atherosclerosis. For example, a recent study showed that liver X receptor (LXR) agonists selectively increased the expression of IL1B in atheroma plaque homogenates-treated human macrophages, revealing that cholesterol and oxysterols promote the occurrence and development of carotid atherosclerosis by inducing IL1B to induce inflammation [33]. Another study showed that ferric ammonium citrate (FAC) could induce the decrease of foam cell activity, inhibit the expression of GPX4 and SIRT1, and increase the level of lipid ROS and the expression of IL-1B [36]. We suggest that ferroptosis of cells increases the expression of the IL-1B gene by inhibiting GPX4 and SIRT1, which activates the inflammatory response, produces a large amount of ROS, and finally leads to the occurrence of carotid atherosclerosis.

The present study has several limitations. First, although we performed a rigorous bioinformatics analysis, we lacked confirmation from experimental and clinical trials, which we will further confirm in future studies to ensure the accuracy of the results. Second, the sample size we analyzed was relatively small, and we need to continue to collect data to expand the sample size to confirm our conclusions. Third, we did not experimentally investigate the potential mechanisms of these genes in carotid atherosclerosis, so further studies are needed in the future.

## 5. Conclusion

In summary, by bioinformatics analysis of the GSE43292 dataset, we identified 33 potential ferroptosis-related genes of carotid atherosclerosis. Moreover, by constructing the PPI network and identifying key modules, 10 genes were identified as key ferroptosis-related genes in carotid atherosclerosis. Among them, IL1B and HMOX1 are possible biomarkers or targets of ferroptosis and carotid atherosclerosis. This study provided some key clues for future research on the molecular mechanism of carotid atherosclerosis and deserves our further study.

## Figures and Tables

**Table 1:** The 33 differentially expressed ferroptosis-related genes in atheroma plaque samples compared to healthy samples.

Gene symbol	log FC	Changes	*P* value	Adj. *P* value	Probe_id
DPP4	1.61087280	Up	2.326828e-07	1.221585e-05	8056222
PGD	0.94074198	Up	3.097850e-07	1.350161e-05	7897620
HMOX1	1.41950225	Up	4.756812e-07	1.591794e-05	8072678
IDH1	0.60008948	Up	7.253949e-07	2.077267e-05	8058552
CYBB	0.74255000	Up	1.817412e-06	3.982799e-05	8166730
NEDD4L	0.56223098	Up	1.853402e-06	3.982799e-05	8021376
FTL	0.55677195	Up	2.253216e-06	4.175076e-05	8030171
PLIN2	1.18298003	Up	4.299166e-06	7.127565e-05	8160297
MMD	0.58977195	Up	5.534257e-06	8.301386e-05	8016832
CTSB	0.63620946	Up	6.327060e-06	9.013363e-05	8149330
ALOX5	0.75010097	Up	8.742280e-06	9.874828e-05	7927215
TNFAIP3	0.59867530	Up	1.187496e-05	1.168942e-04	8122265
NCF2	0.8693150	Up	1.309790e-05	1.250254e-04	7922773
SLC2A3	0.78378788	Up	2.422898e-05	2.051461e-04	7960865
DDIT4	0.5718084	Up	3.214009e-05	2.410507e-04	7928308
SAT1	0.6848509	Up	3.925423e-05	2.747796e-04	8166469
CAPG	0.8185275	Up	4.568905e-05	3.128706e-04	8053417
IL1B	0.7802422	Up	6.628938e-04	2.677071e-03	8054722
SCD	0.7112506	Up	2.676245e-03	8.028735e-03	7929816
CXCL2	0.6020322	Up	2.964514e-03	8.809642e-03	8100994
TFRC	0.5205731	Up	3.996662e-03	1.144499e-02	8093053
NOX4	-0.8820391	Down	1.185850e-07	8.659491e-06	7950933
ZEB1	-0.6688309	Down	1.229041e-07	9.678699e-06	7926916
SLC2A1	-1.0074981	Down	1.944419e-07	1.221585e-05	8129666
GRIA3	-0.5621344	Down	3.428981e-07	1.350161e-05	8169717
CDO1	-0.6634081	Down	1.461069e-06	3.835305e-05	8113641
LURAP1L	-0.5063125	Down	2.109848e-06	4.153763e-05	8154381
ANGPTL7	-0.6068484	Down	5.415842e-06	8.301386e-05	7897675
CAV1	-0.5424884	Down	6.581186e-06	9.013363e-05	8135594
FADS2	-0.5126222	Down	7.422816e-06	9.410893e-05	7940565
EGFR	-0.5477747	Down	1.177876e-05	1.168942e-04	8132860
PRKAA2	-0.7962694	Down	2.401065e-05	2.051461e-04	7901720
LINC00472	-0.5123006	Down	1.344558e-04	7.430451e-04	8127502

**Table 2:** Functional and pathway enrichment analyses for module genes. The top 3 terms were selected based upon the counts of genes enriched and then sorted by *P*.adjust.

Term	Description	Count	P-value	Adj. *P* value	Genes	Gene ratio	Bg ratio
Biological processes							
GO:0006979	Response to oxidative stress	10	1.916459e-09	3.047170e-06	HMOX1, IDH1, CYBB, ALOX5, TNFAIP3, NCF2, NOX4, ANGPTL7, EGFR, PRKAA2	10/32	444/18862
GO:0062197	Cellular response to chemical stress	9	4.204559e-09	3.342625e-06	HMOX1, CYBB, ALOX5, TNFAIP3, NCF2, NOX4, CAV1, EGFR, PRKAA2	9/32	347/18862
GO:0072593	Reactive oxygen species metabolic process	8	1.694471e-08	8.980695e-06	CYBB, ALOX5, NCF2, DDIT4, IL1B, NOX4, CAV1, EGFR	8/32	281/18862
Cellular component							
GO:0043020	NADPH oxidase	3	7.901755e-07	9.087018e-05	CYBB, NCF2, NOX4	3/31	12/19520
GO:0030139	Endocytic vesicle	6	7.618949e-06	4.380896e-04	DPP4, CYBB, NCF2, GRIA3, CAV1, EGFR	6/31	307/19520
GO:0101002	Fcolin-1-rich	4	2.011593e-04	7.711105e-03	IDH1, CTSB, ALOX5, SLC2A3	4/31	185/19520
Molecular functions							
GO:0016175	Superoxide-generating NAD(P)H oxidase activity	3	7.890044e-07	1.222957e-04	CYBB, NCF2, NOX4	3/32	11/18337
GO:0050664	Oxidoreductase activity, acting on NAD(P)H, oxygen as acceptor	3	2.661993e-06	2.063044e-04	CYBB, NCF2, NOX4	3/32	16/18337
GO:0005506	Iron ion binding	4	1.294260e-04	6.687010e-03	FTL, ALOX5, SCD, CDO1	4/32	150/18337
KEGG pathway							
Hsa04216	Ferroptosis	5	2.091572e-08	1.882415e-06	HMOX1, CYBB, FTL, SAT1, TFRC	5/18	41/8113
Hsa04621	NOD-like receptor signaling pathway	5	3.830373e-05	1.723668e-03	CYBB, CTSB, TNFAIP3, IL1B, CXCL2	5/18	184/8113
Hsa04217	Necroptosis	4	3.507860e-04	1.052358e-02	CYBB, FTL, TNFAIP3, IL1B	4/18	159/8113

## Data Availability

Publicly available datasets were analyzed in this study. This data can be found here: https://www.ncbi.nlm.nih.gov/geo/.
